# Assessment of image quality and dose calculation accuracy on kV CBCT, MV CBCT, and MV CT images for urgent palliative radiotherapy treatments

**DOI:** 10.1120/jacmp.v17i2.6040

**Published:** 2016-03-08

**Authors:** Mareike Held, Florian Cremers, Penny K. Sneed, Steve Braunstein, Shannon E. Fogh, Jean Nakamura, Igor Barani, Angelica Perez‐Andujar, Jean Pouliot, Olivier Morin

**Affiliations:** ^1^ Department of Radiation Oncology University of California San Francisco CA USA; ^2^ Department of Radiation Oncology Universitätsklinikum Schleswig‐Holstein Lübeck Germany

**Keywords:** dose calculation, palliative radiation therapy, IGRT, MV CBCT, kV CBCT

## Abstract

A clinical workflow was developed for urgent palliative radiotherapy treatments that integrates patient simulation, planning, quality assurance, and treatment in one 30‐minute session. This has been successfully tested and implemented clinically on a linac with MV CBCT capabilities. To make this approach available to all clinics equipped with common imaging systems, dose calculation accuracy based on treatment sites was assessed for other imaging units. We evaluated the feasibility of palliative treatment planning using on‐board imaging with respect to image quality and technical challenges. The purpose was to test multiple systems using their commercial setup, disregarding any additional in‐house development. kV CT, kV CBCT, MV CBCT, and MV CT images of water and anthropomorphic phantoms were acquired on five different imaging units (Philips MX8000 CT Scanner, and Varian TrueBeam, Elekta VersaHD, Siemens Artiste, and Accuray Tomotherapy linacs). Image quality (noise, contrast, uniformity, spatial resolution) was evaluated and compared across all machines. Using individual image value to density calibrations, dose calculation accuracies for simple treatment plans were assessed for the same phantom images. Finally, image artifacts on clinical patient images were evaluated and compared among the machines. Image contrast to visualize bony anatomy was sufficient on all machines. Despite a high noise level and low contrast, MV CT images provided the most accurate treatment plans relative to kV CT‐based planning. Spatial resolution was poorest for MV CBCT, but did not limit the visualization of small anatomical structures. A comparison of treatment plans showed that monitor units calculated based on a prescription point were within 5% difference relative to kV CT‐based plans for all machines and all studied treatment sites (brain, neck, and pelvis). Local dose differences >5% were found near the phantom edges. The gamma index for 3%/3 mm criteria was ≥95% in most cases. Best dose calculation results were obtained when the treatment isocenter was near the image isocenter for all machines. A large field of view and immediate image export to the treatment planning system were essential for a smooth workflow and were not provided on all devices. Based on this phantom study, image quality of the studied kV CBCT, MV CBCT, and MV CT on‐board imaging devices was sufficient for treatment planning in all tested cases. Treatment plans provided dose calculation accuracies within an acceptable range for simple, urgently planned palliative treatments. However, dose calculation accuracy was compromised towards the edges of an image. Feasibility for clinical implementation should be assessed separately and may be complicated by machine specific features. Image artifacts in patient images and the effect on dose calculation accuracy should be assessed in a separate, machine‐specific study.

PACS number(s): 87.55.D‐, 87.57.C‐, 87.57.Q‐

## I. INTRODUCTION

Modern linear accelerators offer the capability to use on‐board imaging for patient position verification. The intended purpose of the imaging system for 3D pretreatment verification is consistent across linac platforms;^(1)^ however, implementation of different imaging systems is fundamentally different with advantages and disadvantages inherent in each system. Imaging systems differ in the X‐ray source energy used (kV or MV), the acquisition technique (cone beam (CB) or fan beam (FB)), and the reconstruction algorithm.^(2)^ Image quality of these systems has improved significantly over time,^(1)^ presenting the opportunity to use the on‐board imaging systems not only for patient alignment but also for dose verification. Numerous studies have explored the dose calculation suitability of these systems,^3^, ^4^, ^5^, ^6^, ^7^ including studies that focus on rapid planning with palliative intent.^8^, ^9^ For adaptive radiotherapy (RT), some studies combined information from on‐board images and CT images and calculated the dose on the resulting modified image that contained kV CT Hounsfield units (HU).^(10)^

**Figure 1:**
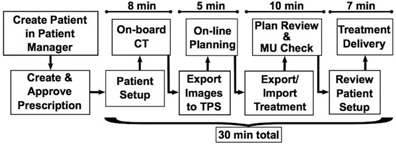
Outline of the workflow for urgent radiotherapy treatments. Approximate times per steps are indicated above each section.

We developed a novel workflow for urgent treatments that consists of patient setup and on‐board CT imaging on the treatment machine, simple planning based on that image set, and treatment delivery (Fig. 1). This workflow is in clinical use on our linac used for on‐call radiotherapy. The current study evaluated dose calculation accuracies for simple plans (one or two beams) on four different treatment machines to determine whether or not each imaging system would be suitable for our urgent treatment workflow.

The aim of palliative therapeutic radiotherapy and the significance of external beam radiotherapy with palliative intent are given in the literature.^11^, ^12^

## II. MATERIALS AND METHODS

### A. Phantoms

Five different site setups were evaluated using two water phantoms and two anthropomorphic phantoms. For Setup 1, we used a 17 cm diameter and 25 cm in long plastic cylinder. Setup 2 was designed to mimic a pelvis made from acrylic sheets bent into a rounded container measuring 38 cm×25 cm×25 cm. Setup 3 utilized the head section of a sliced anthropomorphic phantom, and Setup 4 used the thorax section of the same phantom. Setup 5 was a solid anthropomorphic pelvis phantom.

### B. Imaging systems

Four on‐board imaging systems were evaluated and compared to conventional CT: kV CBCT on the TrueBeam (Varian, Palo Alto, CA), kV CBCT on the VersaHD (Elekta, Crawley, UK), MV CBCT on the Artiste (Siemens, Munich, Germany), and MV CT on the TomoTherapy (Accuray, Sunnyvale, CA). Each imaging system had its own artifacts, and vendors provided different choices of filters for reconstruction, usually based on the treatment site and size. These imaging protocols were used as suggested by the vendors and no additional in‐house filters or image corrections were applied.

#### B.1 kV CBCT

Two different linear accelerators were used for kV CBCT image acquisition. The Varian TrueBeam provided a different imaging protocol for each treatment site. Small phantoms were imaged using a full‐fan and half‐trajectory setting. For head‐sized phantoms, 100 kV tube voltage and 150 mAs tube current were recommended. The thorax protocol specified a half‐fan and full trajectory to increase the field of view up to 45 cm in diameter, using 125 kV and 270 mAs. The pelvis protocol used the same settings with a higher tube current of 1080 mAs. Beam collimation was accomplished with dynamic X and Y jaws.^(13)^ The reported weighted CT dose index (${\text{CTDI}}_{w}$) was between 0.29 and 1.43 cGy per acquisition.

Similarly, the VersaHD provided different protocols based on the treatment site and size. Each protocol used a matching collimator cassette, similar to previous Elekta linac models and described in prior reports.^(13)^ The reconstructed fields of view (FOVs) were up to 27 cm, 41 cm, or 50 cm, depending on the lateral flat panel offset, which can be set to small (S, no offset), medium (M, 11.5 cm lateral offset), and large (L, 19 cm lateral offset).^(13)^ Here, three different protocols were used for phantom image acquisitions: “Head and Neck S20” for the cylinder and head phantom (100 kV and 10 mA, no filter), “Chest M20” for the thorax phantom, and “Pelvis M20” (both 120 kV and 40 mA, with bow‐tie filter) for the water pelvis and anthropomorphic pelvis phantom. The letter S, M, or L denotes the amount of lateral flat panel offset, the number 20 indicates the scan length in Z direction in cm. The physical bow‐tie filter is a filtration cassette that “can significantly change the x‐ray beam spectrum”.^(13)^ Nominal scan doses were reported between 0.12 and 2.20 cGy (Elekta Instructions for use, XVI R4.5)

#### B.2 MV CBCT

MV CBCT images were acquired on a Siemens Artiste that was equipped with the In‐Line kView system. Instead of using the treatment beam, it used a low MV energy and a carbon target during imaging to improve image contrast.^(14)^ Images were acquired using between 5 and 15 monitor units (MUs) (about 5‐15 cGy per acquisition). Imaging protocols were chosen based on the phantom size. For small objects (size of a head), the regular field of view (rFOV) was sufficient to capture the entire anatomy and the system used a half‐arc of 200°. For larger objects (the size of a thorax or pelvis), the extended field of view (eFOV) was used. This mode used a 5.5 cm lateral flat‐panel offset during the acquisition and a 360° gantry rotation. Similar to the other machines, one of three different imaging protocols was chosen based on the treatment site.

#### B.3 MV CT

MV CT images were acquired on the Accuray TomoTherapy machine. Unlike CBCT, it acquires axial image slices on a ring gantry while the patient is translated through the treatment bore. This makes the system extremely stable and prevents reconstruction artifacts.^(2)^ The imaging parameters during image acquisition were set to a normal pitch with a 2 mm reconstruction interval and a dose rate of 45 MU/min for all phantoms, which results in about 1 to 2 cGy dose to the patient per acquisition.^(2)^

#### B.4 Diagnostic kV CT

Standard treatment planning CT images were acquired on the diagnostic kV CT scanner MX8000 by Philips (Philips Healthcare, Andover, MA). All phantoms were imaged using 120 kV tube voltage, 209‐244 mA tube current and a slice thickness of 2 mm. The delivered dose per scan was about 0.20 cGy. Treatment plans based on these diagnostic CT images were used as the reference to evaluate the treatment plans based on the four on‐board systems described above.

### C. Image noise, contrast, uniformity, spatial resolution

Image quality, noise, contrast‐to‐noise ratio (CNR), and uniformity (UN) were assessed for phantom images on each machine, using the definitions:

(1) $$Noise=100*\frac{\sigma_{\left(Water\right)}}{\mu_{\left(Water\right)}},$$

(2) $$CNR=\frac{\mu_{\left(ROI_{bone}\right)}-\mu_{\left(Water\right)}}{\sigma_{\left(Water\right)}},$$

and

(3) $$UN=100*\frac{\mu_{\left(Water_{periphery}\right)}-\mu_{\left(Water_{center}\right)}}{\mu_{\left(Water_{pelvis}\right)}}.$$

Regions of interest (ROIs) were drawn to obtain the mean image value for water, $\mu_{\left(Water\right)}$, and bone $\mu_{\left(ROI_{bone}\right)}$, and the standard deviation (SD) $\sigma_{\left(Water\right)}\cdot \;\mu_{\left(Water_{periphery}\right)}$ included the mean value of the ROI on the lateral edges of the pelvic water phantom. $\mu_{\left(Water_{center}\right)}$ was the mean value of the ROI in the center of the same phantom, and $\mu_{\left(Water_{pelevis}\right)}$ was the mean image value of the ROI covering the entire area inside the phantom.

The spatial resolution of each system was defined using the Catphan504 phantom (The Phantom Laboratory, Salem, NY) with the CTP528 high resolution module, which contains resolution sections ranging from 1‐21 lps/cm. The phantom was imaged on all five imaging systems, using the same image protocol as the one that was used for the anthropomorphic head phantom. The modulation transfer function (MTF) was calculated for each machine and the 50% critical frequency (50% cf) was defined for a comparison of the spatial resolution among all systems.

### D. Density calibration

Initial attempts to use a single image value to density calibration phantom across all five machines failed. Thus, phantom images acquired on each machine were used to calibrate the physical density to the image values, as required by the treatment planning system. ROIs were drawn for different densities on each image set. The mean HU value of each ROI was assigned to the according density of the same ROI on the kV CT image. A separate image value to density calibration (IVDC) was created for each image acquisition protocol.

Accurate dose calculation requires consistent IVDCs all throughout the phantom. Nonuniformity could cause objects to appear more or less dense than they actually were in parts of the images. Before using the IVDCs for dose calculation, they were used to display the object in density values, highlighting areas in which artifacts changed the density value of the object.

### E. Dose planning on phantom images

A simple treatment plan was created on each image set (Table 1). For Setup 1 and 2, a single posterior‐anterior (PA) beam with an open field was planned. Two opposed beams were used for Setups 3 to 5. Each plan used two different prescriptions. In prescription 1, a set number of MUs were prescribed. In prescription 2, a dose to a point at mid‐plane was prescribed. All calculated plans were compared to the plan on the standard kV CT. Prescription 1, with a set number of MUs, was used to compare the dose distribution within the phantoms. The mean dose difference and gamma index for 3%/3 mm criteria were assessed, including all values above the low‐dose threshold of 30% of the maximum dose. Prescription 2 evaluated the plan dose by looking at the total number of MUs that were prescribed resulting from treatment plans based on kV CBCT, MV CBCT, or MV CT images compared to kV CT images.

**Table 1:** Difference in calculated dose for prescription 1 relative to the treatment planning CT

	*kV CBCT (True Beam)*		*kV CBCT (Versa)*		*MV CBCT (Artiste)*		*MV CT (Tomo)*	
*Phantom*	*γ‐index* (3%/3mm)	*Mean (%) SD*	*γ‐index* (3%/3mm)	*Mean (%) SD*	*γ‐index* (3%/3mm)	*Mean (%) SD*	*γ‐index* (3%/3mm)	*Mean (%) SD*
Water	97.28	−0.28	97. 20	−0.59	96. 74	−0.71	97. 03	−0.44
Cylinder		2.38		2.69		6.46		3.71
Water	99.37	0.27	99.35	−0.05	99.13	0.21	100.00	−0.29
Pelvis		0.80		0.82		5.42		0.63
Head	94.36	−1.93	99.67	−1.15	96.70	−3.74	99.47	−1.73
		4.28		6.25		12.52		6.08
Neck	99.62	0.64	97.88	−3.54	99.51	−0.32	99.25	−0.96
4.99		15.15		6.78		8.99	
Hip	99.99	−0.15	99.81	0.59	99.86	−0.22	99.99	0.18
4.74		2.37		3.78		4.92	

## III. RESULTS

### A. Image noise, contrast, uniformity, spatial resolution

Visually, image quality was sufficient for emergency type treatment plans on all machines and bony anatomy was displayed with enough contrast to define its structure. Among all phantom images in this study, MV CT images had the highest noise level, six times higher than the noise in the kV CT images. MV CBCT images showed the lowest CNR between bone and water. kV CBCT images on the VersaHD overcorrected the image nonuniformity such that the image center appeared at a higher image value than on the edges of the image. Similarly, but less pronounced, MV CBCT images showed image nonuniformity with slightly lower image values left and right of the image center. Out of all five imaging systems, spatial resolution was worst on Artiste images, with a 50% critical frequency about 2.5 times less than for kV CT and TrueBeam kV CBCT images. The dose per scan was comparable on all imaging systems, except the Artiste. Here, dose was up to seven times higher for eFOV acquisitions. All values describing image quality are summarized in Table 2 for comparison.

**Table 2:** Image dose, noise, CNR, uniformity, and spatial resolution

	${\text{CTDI}}_{vol}$ *(cGy) rFOV/eFOV*	*Noise*	*CNR (bone/water)*	*Uniformity*	*Spatial Resolution 50% cf (1/cm)*
kV CT (MX 8000)	0.20	0.53	161.5	0.1	4.1
kV CBCT (TrueBeam)	0.29/1.43^a^	2.10	52.2	−1.0	4.1
kV CBCT (Versa)	0.12/2.20^a^	3.07	36.7	6.7	2.1
MV CBCT (Artiste)	5.00/15.00^b^	1.91	14.9	−4.5	1.6
MV CT (Tomo)	∼2.00 ^b^	3.14	15.7	0.0	2.1

a ^a^ ${\text{CTDI}}_{w}$, dose

### B. Density calibration

**Figure 2:**
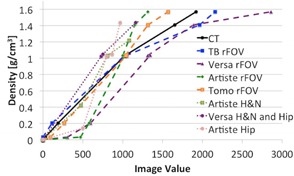
IVDC curves for kV CT and on‐board imaging systems. The VersaHD required two separate curves for rFOV and eFOV imaging protocols. The Artiste required three protocols, one for rFOV and two for eFOV.

In previous studies, Thomas^(15)^ reported a resulting dose difference of 1% for an electron density difference of 8% for typical radiotherapy beams, and Hatton et al.^(16)^ similarly showed that 21% difference in electron density resulted in 2.6% dose difference. Thus, IVDCs for the same machine that were within 8% difference in density were combined into one curve. For the CT, TrueBeam and TomoTherapy units, all calibration points were within 8% of each other, which resulted in less than 1% dose difference according to Thomas.^(15)^ Thus, the TrueBeam and Tomotherapy units required only one IVDC each, despite using different imaging protocols. The Versa required two different curves, one for small objects (such as the head) and one for large objects (such as the thorax or pelvis). The Artiste was assigned three calibrations, which included separate IVDCs for images of the head, thorax, and pelvis. In the end, eight different IVDCs were entered into the treatment planning system for five different machines. All IVDC curves are plotted in Fig. 2.

**Figure 3:**
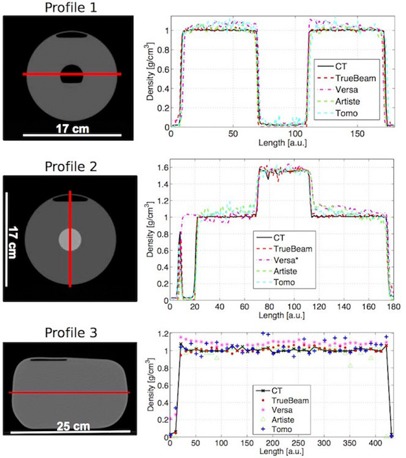
Density profiles for the cylindrical and the pelvic water phantom. (left) CT image slice of the water phantoms. The red line indicates the path of the profile. (right) Density profiles for the acquired CT on each imaging system. The HU values are converted to density values using the machine‐specific IVDC. (*The air bubble in the cylindrical phantom was removed before image acquisition on the VersaHD.)

Using the resulting IVDCs, the images were converted into physical density. Figure 3 shows a density profile (solid line) for the water cylinder and the pelvic water phantom. The same profile was plotted for all imaging machines studied here. The image noise in MV CT images was clearly visible. Versa images showed an inconsistency in image value to density conversion in the image center in case of the water cylinder phantom. This appears to be due to the image nonuniformity.

Table 3 lists a summary of the mean density difference in percent relative to the kV CT density along each profile and one standard deviation.

**Table 3:** Density difference along image profiles from Fig. 3. Values are the relative difference to kV CT in percent

	*Profile 1*	*Profile 2*	*Profile 3*
kV CBCT (TrueBeam)	0.70±1.38	0.71±2.13	0.85±3.32
kV CBCT (Versa)	4.86±3.32	3.99±6.61	8.05±2.77
MV CBCT (Artiste)	0.80±2.74	4.76±6.83	0.06±3.73
MV CT (Tomo)	3.30±4.49	3.01±4.69	3.40±7.00

### C. Dose calculation accuracy

**Figure 4:**
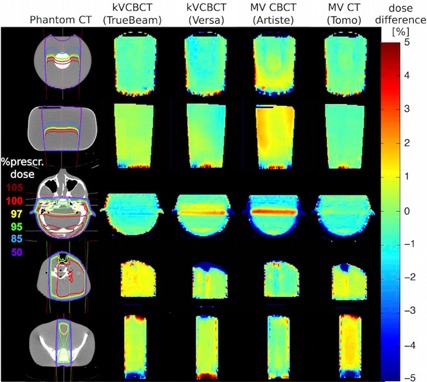
Dose difference maps. Percentage difference of simple dose plans based on kV CBCT, MV CBCT, and MV CT images compared to diagnostic kV CT‐based images. The left column bar shows the dose distribution within the CT image as percentage of the maximum dose. The columns to the right show the relative percentage differences where green is no difference, blue is underdosing, and red is overdosing on the linac's on‐board images.

Based on prescription 1, local dose calculation errors were identified. Figure 4 summarizes the percentage dose calculation differences in a color map. The left column shows the diagnostic CT center slice of each phantom with the planned dose distribution in percent, relative to the maximum dose. The four columns to the right map the local dose differences relative to the kV CT plan for the according image slice, which resulted from the treatment plan based on the onboard images of all four systems. The color map is scaled from −5% (blue) difference to +5% (red) difference, with green indicating good agreement between both plans. The calculated dose differences, expressed by the gamma index with 3%/3 mm criteria, the overall mean difference, and standard deviation, are summarized in Table 1.

Another approach to evaluate the outcome of the treatment plans was the comparison of MUs per plan. These were obtained based on the treatment dose prescribed to a point inside the phantom. In all cases, calculated MUs were within 5% of the number of MUs for the same kV CT‐based plan. The relative differences of MUs for each imaging system and treatment site are listed in Table 4.

**Table 4:** Percentage difference in MUs compared to the kV CT plan prescribed to a point at mid‐plane

	*kV CBCT (TrueBeam)*	*kV CBCT (Versa)*	*MV CBCT (Artiste)*	*MV CT (Tomo)*
Water Cylinder	−0.82	2.46	0.82	0.00
Water Pelvis	−0.13	0.00	−0.93	0.66
Head	2.35	−0.59	−0.59	0.00
Neck	−0.62	−1.23	1.23	−1.23
Hip	−3.02	−2.63	−2.10	−3.15

## IV. DISCUSSION

Despite the reported differences in image quality, each machine produced sufficient image contrast to identify bony anatomy, which is important for defining the treatment target in many emergency setups. Further reduction in imaging dose is not necessary on the TrueBeam and VersaHD, since imaging protocols are preset and already optimized for low dose delivery.

Imaging dose for the TomoTherapy could possibly be lowered by using a 3 mm pitch instead of 2 mm. Reducing the MUs per MV CBCT acquisition on the Artiste may also be possible, if requested. However, in both cases, it is important to ensure this would not affect the IVDC.

In case of the VersaHD and Artiste, the IVDCs obtained were dependent on the image protocol and object size. The TomoTherapy and TrueBeam systems produced spatially stable image values regardless of these two factors. For the Artiste, all image value to density calibrations have been tested for stability over time before clinical implementation. This remains to be done for all other systems.

Although the contrast to noise ratio and spatial resolution for Artiste images were about a tenth and a third less than that of kV CT images, respectively, MV CBCT images were still adequate for simple treatment plan dose calculation. Overall, this study on water and anthropomorphic phantoms showed that image acquisition on all four on‐board imaging systems provided acceptable dose calculation accuracy for simple treatment plans of single or opposed beams in case of head, head and neck, and pelvis treatments. Prescription 1 revealed areas of local dose differences of up to 5% within the phantom, showing the largest dose differences in MV CBCT‐based treatment plans. Local differences of more than 5% were observed only on the field edges, irrespective of the field size. Our recommendation based on these observations is that areas of relative differences >5% should be avoided when choosing a prescription point.

Prescription 2 was used as an additional test to determine the overall difference in treatment plans. Based on a dose prescription to a point at mid‐plane, the total number of MUs was within the objective of ±5% relative to the kV CT‐based plan for all imaging machines and all treatment sites. In the end, this would be the difference in delivered dose for these treatments. Nevertheless, knowledge of where dose calculation may be less accurate was important to correctly prescribe the treatment dose and make judgments regarding dose distribution.

With this accuracy, all treatment fractions could be delivered using this setup and treatment plan. However, this should be decided on a case‐by‐case basis, taking into account patient‐specific factors and treatment plan details.

**Figure 5:**
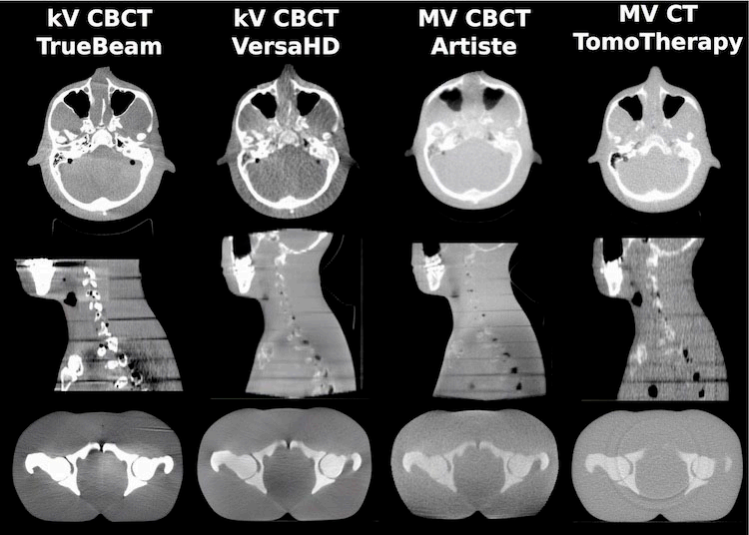
CT images of the phantom used in Setup 3, 4, and 5.

**Figure 6:**
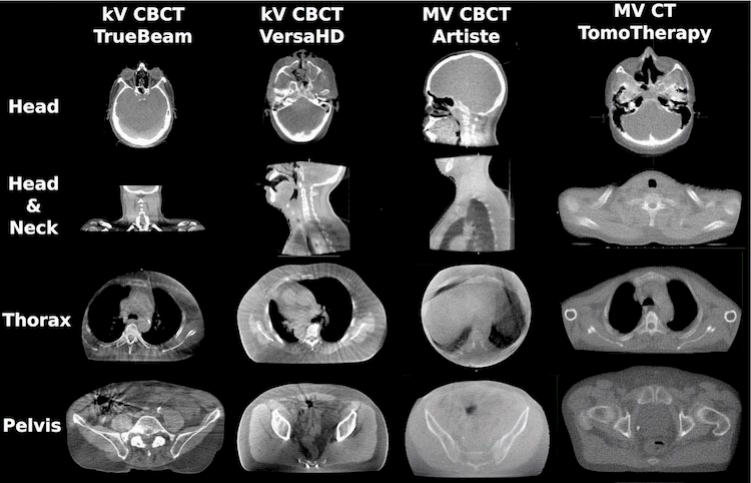
Patient CT images of different treatment sites. The images were acquired using the indicated machines’ on‐board imaging system.

Figure 5 shows a CT slice of the phantoms used in Setups 3 to 5, which were acquired using each of the linac's on‐board imaging system. In comparison, Fig. 6 is a collection of patient images. These images demonstrate that the phantoms used provided good representation of realistic image quality. Many of the image artifacts could be observed in phantom as well as patient images. For example, nonuniformity caused by the transition from the neck to the shoulders was present in all cone‐beam images. Images using energies in the MV range showed a much lower CNR than kV images. This is mainly due to the dominant Compton effect for MV energy photon interaction with matter, in which case photon attenuation is independent of the atomic number Z. For photons with energies in the kV range, photoelectric attenuation is dominant, which is proportional to Z^3^, resulting in higher contrast, especially for soft tissues. kV CBCT images presented brighter shades of gray‐level around bony anatomy. Nevertheless, artifacts caused due to organ motion are not captured in phantom images. Consequently, streaking artifacts were much more pronounced in pelvic images of actual patients than in the phantom images. A separate study that compares treatment plans based on patient images of each of the on‐board imaging systems to the same plans based on the kV CT would be advised. Also of interest may be another study that investigates artifacts specific to patients with metal implants or prostheses.

### A. Field of view

The field of view remained a limitation for this application. In case of the TrueBeam, the 15 cm scan length might be insufficient to capture the anatomy for a whole brain treatment. Artiste images were limited to $31\times 31\;{\text{cm}}^{2}$ for an axial FOV, which was sufficient in most cases; however, opposed beam planning might present a problem for large patients. The VersaHD allowed a FOV up to $50\times 50\;{\text{cm}}^{2}$ for axial slices when using the largest lateral flat panel offset, but image uniformity was reduced compared to the medium FOV size of $42\times 42\;{\text{cm}}^{2}$, which was used here. Additionally, in all cases, the dose calculation accuracy is diminished towards the edges of the image. Consequently, it is important that the patient setup point and image isocenter were in close proximity to the treatment isocenter.

### B. Clinical implementation

Another potential limitation is that DICOM export to the treatment planning system might require extra time if it was not integrated into the linac software. This may depend on the combination of linac and patient management software used in the clinic. Furthermore, MV CT acquisition times on the TomoTherapy were known to extend the duration of the workflow by a few minutes. Upon clinical implementation, a procedure that ensures the choice of the correct IVDC within the treatment planning system is strongly recommended.

Previous research reported on a similar workflow for palliative treatments on the TomoTherapy linac, using the commercial software StatRT, which was created specifically for this purpose on the TomoTherapy.^9^, ^17^, ^18^, ^19^ However, this software was not available in our clinic at the time of this study.

A summary of clinically important factors for each machine is provided in Table 5.

The dosimetric prerequisites to accurately and rapidly simulate, plan, and treat were given on each machine studied. Nevertheless, initial individual dose verification using patient images instead of phantoms is still recommended for each machine.

This study compared dose calculations for treatment sites of the head, neck, and pelvis. Treatments of the thorax specifically were not studied here, as rigid phantoms seemed inappropriate for the purpose. In those particular cases, the main challenge would be artifacts caused by tissue motion during the image acquisition, which does not occur in rigid phantoms and in some cases the need for sufficient image quality to count vertebral bodies reliably. A collection of patient images for each machine will be required before making any qualified recommendations on dose calculation accuracy around and within lung tissue.

**Table 5:** Summary of clinically important factors for each on‐board imaging system

	*kV CBCT (TrueBeam)*	*kV CBCT (Versa)*	*MV CBCT (Artiste)*	*MV CT (Tomo)*
Multiple IVDC calibrations necessary?	no	yes	yes	no
Mean dose calculation accuracy <5%/<10% including 1 SD?				
Head	no/yes	no/yes	no/no	no/yes
Neck	no/yes	no/no	no/yes	no/yes
Pelvis	yes/yes	yes/yes	yes/yes	no/yes
Difference of prescribed MU to mid‐plane <5%?	yes	yes	yes	yes
Max. field of view (diameter, length (cm))	45, 15	50, 27	31, 25	40, 26^c^
Acquisition & reconstruction time	<2min	<2min	<2min	∼5min ^a^

a ^a^ Scan length variable ‐ acquisition time estimated for 26 cm scan length.

## V. CONCLUSIONS

On‐board imaging provided a useful tool to simulate patients in urgent treatment situations for a simplified and streamlined treatment procedure. Although implementation of the workflow may involve additional work, the prerequisites for dose calculation based on on‐board images were given. With machine‐specific IVDCs, the calculated MUs per plan were within the objective of ±5% difference relative to kV CT‐based planning. Local dose differences were identified for three treatment sites (head, neck, and pelvis).

In contrast to urgent hand calculation based treatments, if CT‐simulation is unavailable outside of regular work hours, this approach offers an enormous advantage through 3D CT‐based treatment planning that makes use of modern digital capabilities. Compared with the challenges of expedited kV CT‐based urgent plans, the workflow suggested here reduces patient waiting and setup times and provides a predictable treatment timetable, combining simulation, planning, and treatment into one session.

## ACKNOWLEDGMENTS

We would like to thank Atchar Sudhyadhom, Christopher McGuinness and Aaron Garcia, all from UCSF, for their help acquiring the images on the various imaging systems.

## COPYRIGHT

This work is licensed under a Creative Commons Attribution 4.0 International License.
